# Impact of *agr* dysfunction on virulence profiles and infections associated with a novel methicillin-resistant *Staphylococcus aureus* (MRSA) variant of the lineage ST1-SCC*mec* IV

**DOI:** 10.1186/1471-2180-13-93

**Published:** 2013-04-27

**Authors:** Fabienne Antunes Ferreira, Raquel Rodrigues Souza, Bruno de Sousa Moraes, Ana Maria de Amorim Ferreira, Marco Antônio Américo, Sérgio Eduardo Longo Fracalanzza, José Nelson dos Santos Silva Couceiro, Agnes Marie Sá Figueiredo

**Affiliations:** 1 Departamento de Microbiologia Médica, Instituto de Microbiologia Paulo de Góes, Universidade Federal do Rio de Janeiro, Rio de Janeiro, RJ, Brazil; 2 Departmento de Virologia, Universidade Federal do Rio de Janeiro, Instituto de Microbiologia Paulo de Góes Rio de Janeiro, Rio de Janeiro, RJ, Brazil

**Keywords:** MRSA, ST1-SCC*mec*IV, USA400, *agr*, Biofilm, Virulence factors

## Abstract

**Background:**

A novel variant of the ST1-SCC*mec*IV methicillin-resistant *Staphylococcus aureus* (MRSA) lineage, mostly associated with nosocomial bloodstream infections (BSI), has emerged in Rio de Janeiro. Bacterial biofilm has been considered a major virulence factor in central venous catheter-associated BSI. The mechanisms involved in biofilm formation/accumulation are multifactorial and complex. Studies have suggested that biofilm production was affected *in vitro* and vivo for *agr*-null mutants of *S. aureus*.

**Results:**

The impact of naturally occurring inhibition of *agr* signaling on virulence profiles and infections associated with the ST1 variant was investigated. *agr* dysfunction was detected in a significant percentage (13%) of the isolates with concomitant increase in biofilm accumulation *in vitro* and *in vivo*, and enhanced ability to adhere to and invade airway cells. The biofilm formed by these ST1 isolates was *ica*-independent and proteinaceous in nature. In fact, the improved colonization properties were paralleled by an increased expression of the biofilm-associated genes *fnbA*, *spa* and *sasG*. The transcription of *sarA*, a positive regulator of *agr*, was two-times reduced for the *agr*-dysfunctional MRSA. Remarkably, the *agr* inhibition was genetically stable. Indeed, *agr*-dysfunctional isolates succeed to colonize and cause both acute and chronic infections in hospitalized patients, and also to effectively accumulate biofilm in a mouse subcutaneous catheter implant model.

**Conclusion:**

The ability of *agr*-dysfunctional isolates to cause infections in humans and to form biofilm in the animal model suggests that therapeutic approaches based on *agr*-inactivation strategies are unlikely to be effective in controlling human-device infections caused by ST1 isolates. The increased biofilm accumulation associated with the acquisition of multiple antimicrobial resistant traits might have influenced (at least in part) the expansion of this USA400 related clone in our hospitals.

## Background

Community-acquired methicillin-resistant *Staphylococcus aureus* (CA-MRSA) lineage ST1- SCC*mec* IV was first reported in the 1980s among aborigines in Australia (WA-1 clone) and in the USA (MW2/USA400 clone) where cases of fatal infections were reported in Michigan, Minnesota and North Dakota [1-3]. Nowadays, CA-MRSA infections have been described in different countries involving a number of genetically distinct lineages [4,5].

Many CA-MRSA isolates (including USA300, USA400 and USA1100) carry *lukSF* encoding for Panton-Valentine leukocidin (PVL). Despite the controversy regarding the role of the PVL, this leukocidin has been linked to severe skin infections and necrotizing pneumonia [6-8]. In the USA, USA300 has replaced USA400 as the predominant clone in many communities [9]. However, USA400 isolates were the main cause of an outbreak of skin infections that occurred in rural southwestern Alaska, in 1996–2000 [10]. Indeed, USA400 was the far most common CA-MRSA clone recovered from three northern remote communities of Saskatchewan, Canada [11]. In 2005, a novel variant of the lineage ST1-SCC*mec*IV emerged in Rio de Janeiro city as an important cause of bloodstream infections (BSI) [12]. It is intriguing that despite the genetic relationship with Australian WA-1 and MW2/USA400, isolates of this novel clone were PVL-negative, multiresistant and mostly involved in hospital-associated BSI [12]. It is still poorly understood why isolates of CA-MRSA have become successful so quickly [13]. Nevertheless, for hospital-associated MRSA (HA-MRSA), the bacterial ability to produce biofilm has been recognized as an important virulence factor for the pathogenesis of intravenous catheter-related bacteremia and infections associated with the use of medical prosthesis. In addition, the bacterial ability to adhere to, colonize and invade host tissues is considered important factor associated with bacterial virulence, adaptation and spread [14,15]. Different surface proteins have been implicated in biofilm formation/accumulation and host colonization, including fibronectin-binding proteins A and B (FnBPAB), *S. aureus* surface protein G (SasG) and staphylococcal protein A (Spa) [16-19]. In addition, extracellular DNA (eDNA) has also been associated with bacterial biofilms [20].

It is also well known that virulence in *S. aureus* is modulated by an intricate regulatory network [21]. The accessory gene regulator (*agr*), the major *S. aureus* quorum sensing system, down-regulates a number of genes encoding for cell-surface proteins involved in colonization processes, and up-regulates (by an indirect mechanism involving RNAIII dependent down-regulation of Rot) different exoproteins associated with host-cell damages [22]. Previous works have suggested that inactivation of Agr could be very effective at inhibiting *S. aureus* infections [23], including those associated with implantable medical devices [24,25]. Studies have demonstrated that biofilm production*,* host cell adhesion and invasion as well as other mechanisms involved in the establishment and course of staphylococcal diseases were affected by knockout of the *agr* locus [26-28]. Despite the improvements achieved in staphylococcal virulence, most of the investigations have been carried out using relatively few laboratory constructions or clinical isolates [28]. In addition, those results have not been validated using current clinical isolates of MRSA. In this paper we characterized the biofilm formed by USA400-related (ST1-SCC*mec*IV) MRSA emergent in Rio de Janeiro, investigated the adhesive and invasive properties of naturally *agr*-dysfunctional isolates and analyzed the impact of the *agr* inhibition on *S. aureus* infections associated with the use of medical device. Our results suggest that strategies based on *agr* inactivation approaches may not be effective as an anti-biofilm strategy in the management of device-associated infections caused by these MRSA isolates.

## Results

### Biofilm

**Figure 1:**
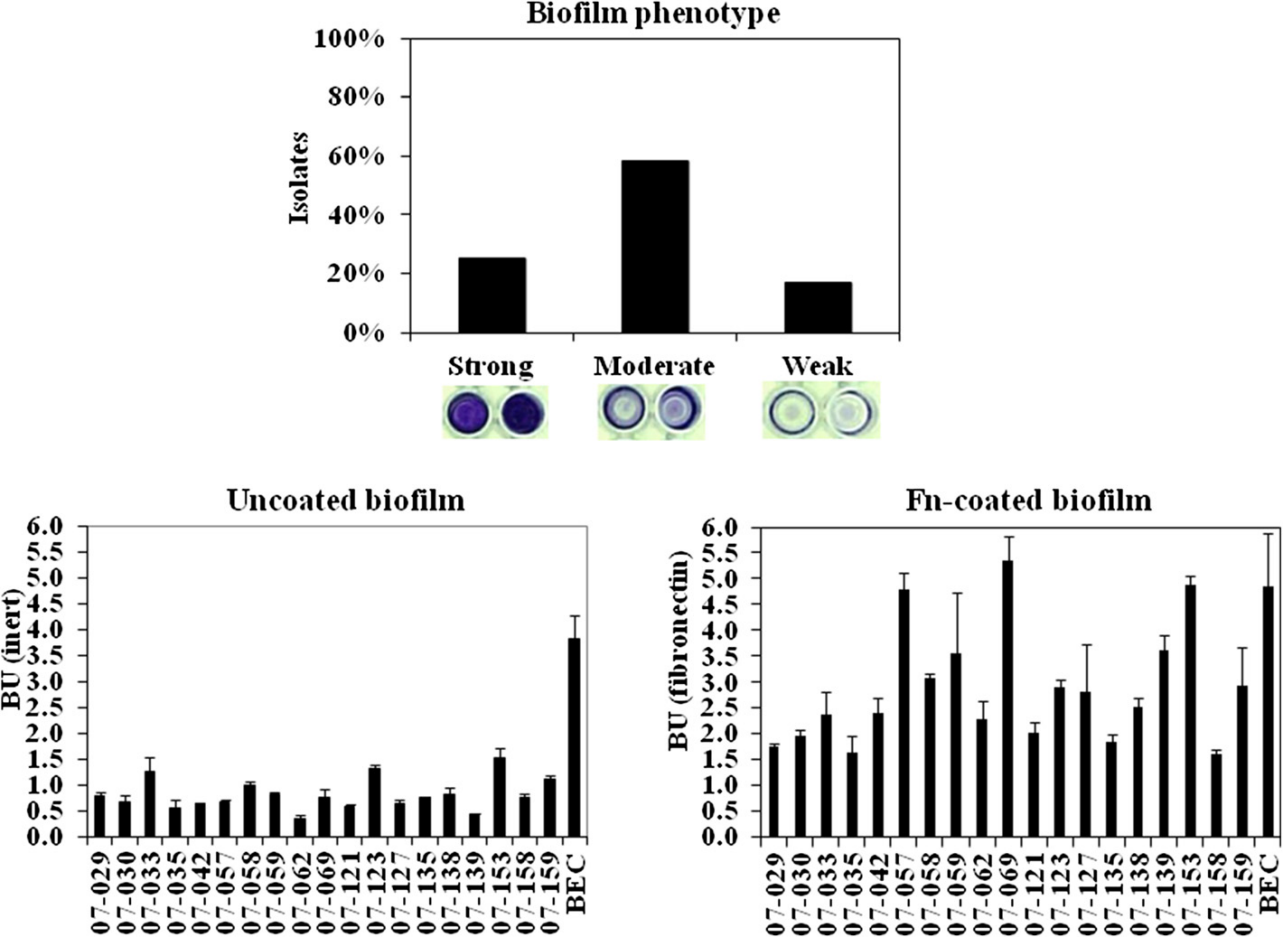
**Biofilm formed by ST1 isolates. *Top:*** Percentage of the total 60 ST1 isolates displaying strong, moderate and weak biofilm phenotypes. Wells show the different biofilm phenotypes formed on inert polystyrene surfaces by representative ST1 isolates. ***Bottom:*** Biofilm formed on inert or fibronectin-coated surfaces by 19 ST1 isolates.

All sixty ST1 isolates tested were able to produce biofilm on inert surfaces. The majority (58.3% and 25%; respectively) exhibited a moderate (BU varying from 0.468 to 0.901) or strong (BU varying from 1.008 to 3.615) biofilm phenotypes (Figure 1, top). For 19 randomly selected isolates, the ability to accumulate biofilm on human Fn-coated surfaces increased significantly (*p*<0.01 to *p*<0.0001) when compared with that on inert surfaces (Figure 1, bottom).

### Proteinaceous nature of the biofilm

**Figure 2:**
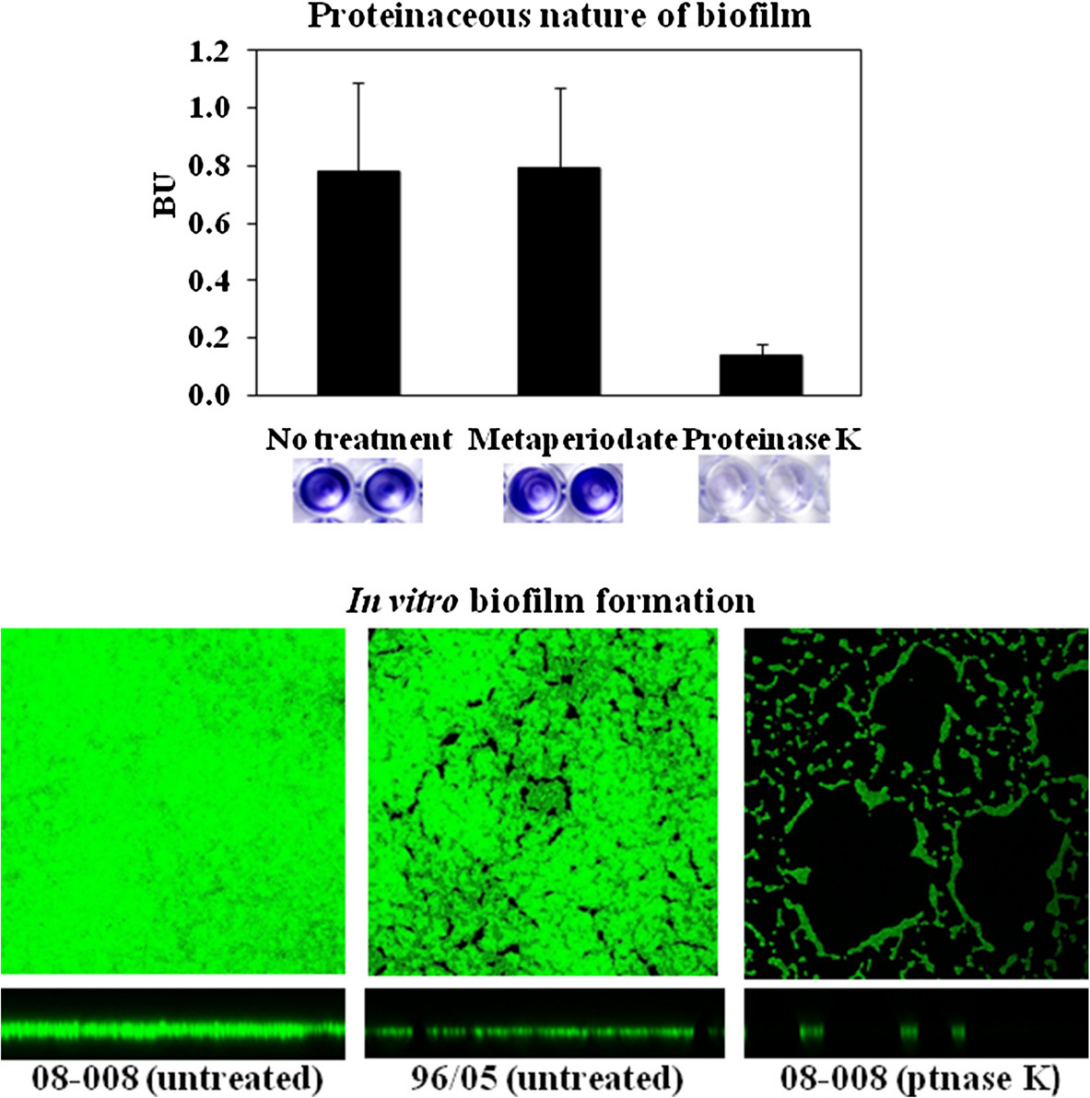
**Proteinaceous nature of the biofilm. *Top:*** Effect of 1mM/well sodium metaperiodate or 6U/well proteinase K on preformed biofilm. Wells show the effect of these compounds on biofilms preformed on inert polystyrene surfaces by representative ST1 isolates. ***Bottom:*** Confocal laser scanning microscopy (CLSM) images of proteinase K-treated and -untreated biofilms stained with SYTO 9. The square indicates the slice of the biofilm from which the XY image was taken. The horizontal bar indicates the location of the X plane from which the cross-section was taken. Isolate 08–008 (strong biofilm producer, *agr*-dysfunctional), 96/05 (moderate biofilm producer, *agr*-functional).

Treatment with proteinase K virtually disrupted preformed biofilms for 12 ST1 isolates tested. However, the carbohydrate oxidant metaperiodate almost did not affect the biofilm accumulated by these isolates (Figure 2, top). CLSM studies revealed that the *agr*-dysfunctional 08–008 accumulated a denser and compact biofilm when compared to the heterogeneous film formed by the *agr*-functional isolate (96/05). Despite the stronger biofilm phenotype displayed by the isolate 08–008, proteinase K could significantly remove the biological film accumulated (Figure 2, bottom).

### Role of eDNA in ST1 biofilm

**Figure 3:**
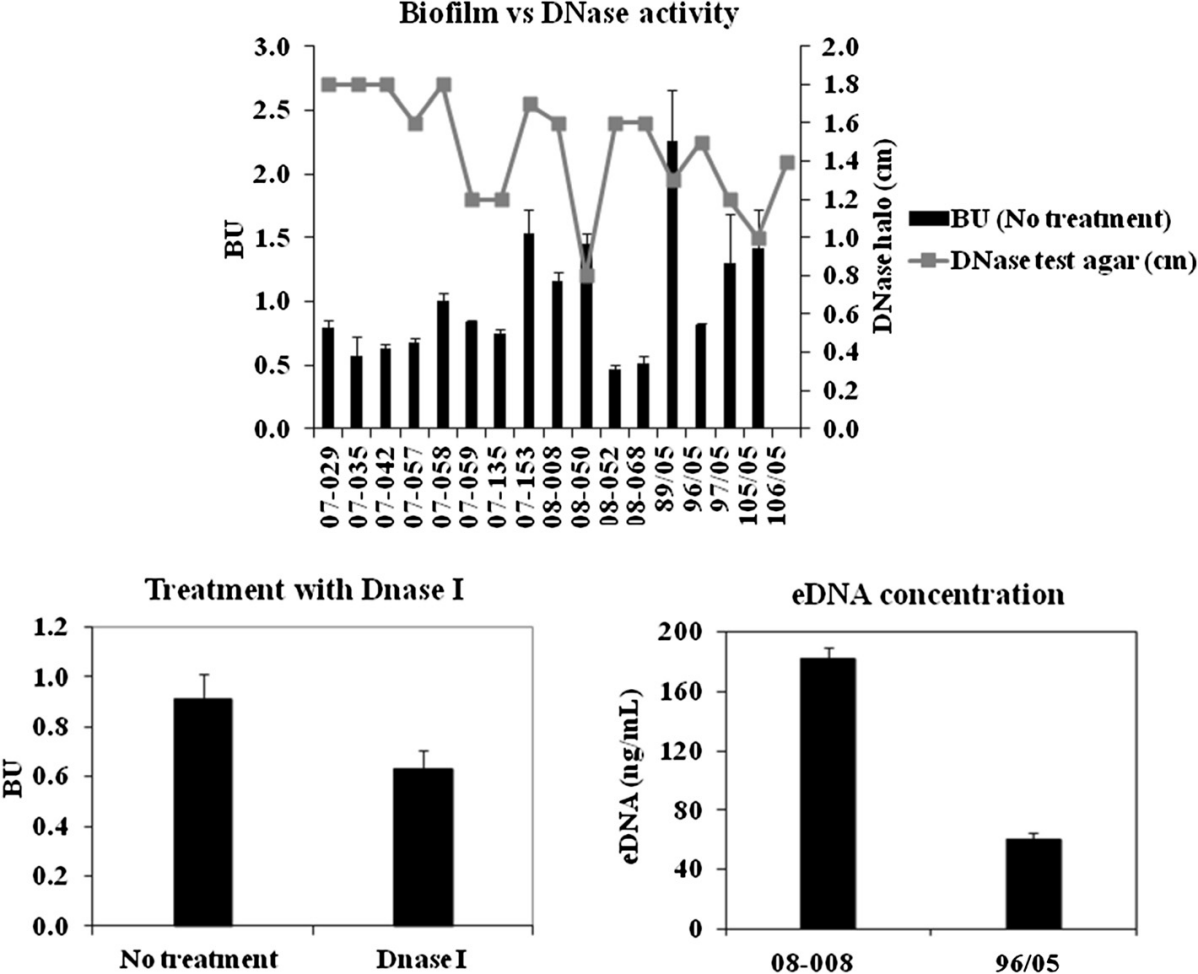
**Bacterial DNase activity, treatment of the biofilm with DNase I and eDNA assay. *Top:*** DNase activity was detected in culture supernatants of 16 ST1 isolates by measuring the halo size (cm) produced on Difco™ DNase Test Agar (BD). BU: Biofilm values for 16 ST1 isolates using inert polystyrene. ***Left bottom:*** For 16 ST1 isolates, 56U/well of DNase I were added to the culture media and the amount of biofilm accumulated determined. ***Right bottom:*** The concentration of eDNA determined in the biofilm supernatant. Isolate 08–008 (strong biofilm producer, *agr*-dysfunctional), 96/05 (moderate biofilm producer, *agr*-functional).

**Figure 4:**
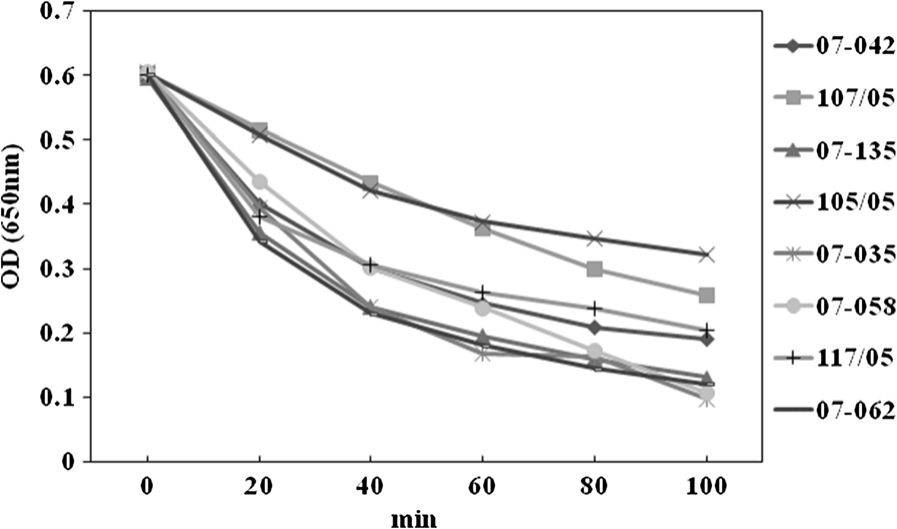
**Autolysis assays for USA400-related isolates.** 07–058, 105/05, 107/05 are strong biofilm producers; 07–035, 07–042, 07–135 moderate; and 07–062, 117/05 weak producers.

No correlation was detected between the activity of bacterial DNase and the levels of biofilm accumulated by 17 USA400-related isolates displaying strong, moderate or weak biofilm phenotypes (Figure 3, top). The addition of 28U/well DNase I in the culture media did not significantly affect the biofilm formed by these ST1 isolates. However, when this concentration was increased to 56U/well, a significant (p=0.0078) reduction of 31% in biofilm accumulation was detected (BU untreated =0.91±0.1 and treated =0.63±0.078; Figure 3, left bottom). In addition, the concentration of eDNA recovered from the supernatant of the strong biofilm producer (BU=1.167 ±0.07) isolate 08–008 was 182 ng/mL, three-times higher than that determined for the weaker producer (BU=0.348±0.01) isolate 117/05 (Figure 3, right bottom). In agreement with these results, we have also detected a moderate correlation (r=0.59) between bacterial autolysis and biofilm accumulation, when 4 stronger biofilm producers were compared with the same number of weaker producers (Figure 4).

### *agr*RNAIII inhibition

**Figure 5:**
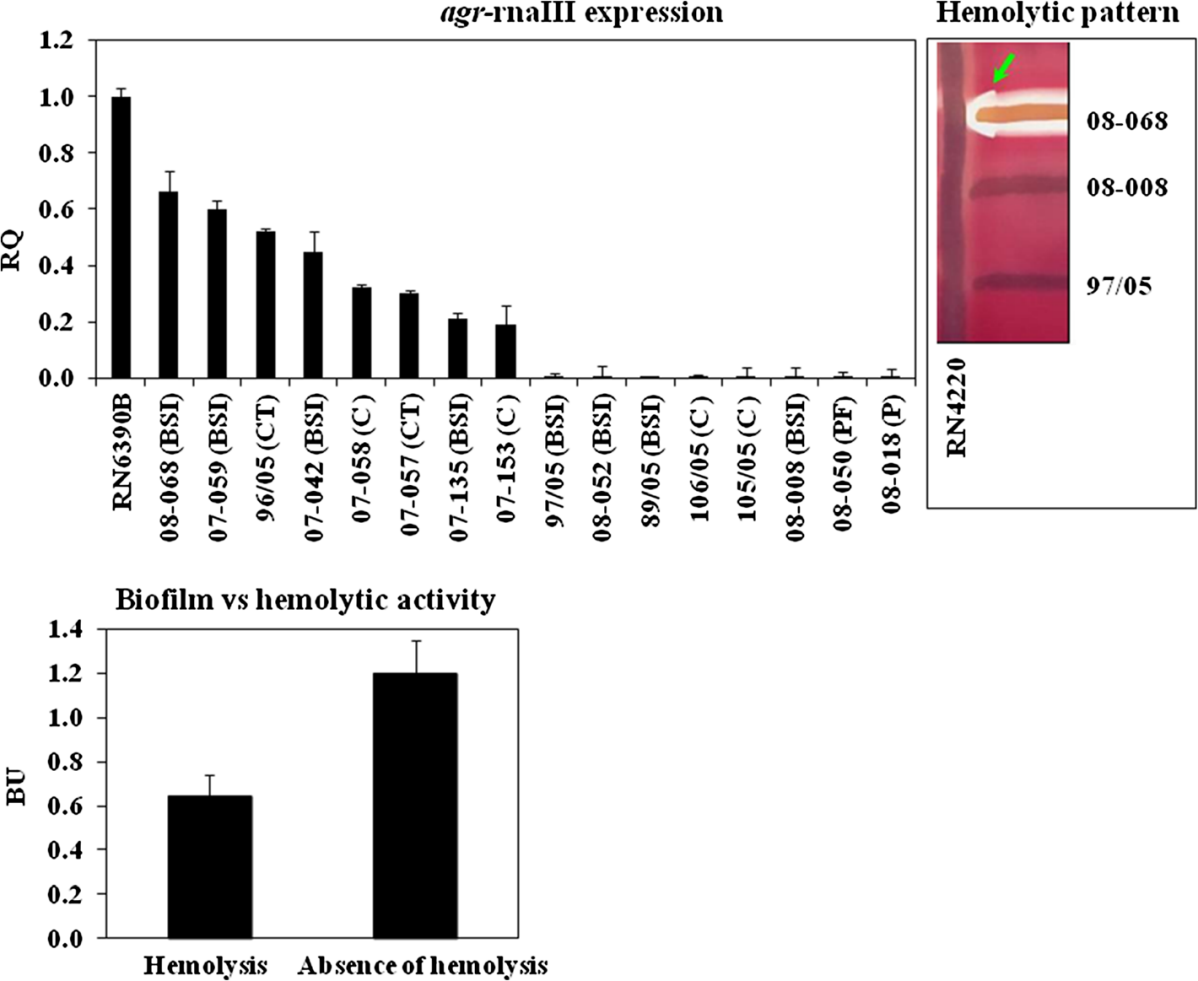
***agr *differential expression in USA400-related isolates. *Top left:****rnaIII* expression was analyzed by RT-qPCR using ΔΔC_T_ comparative method. RQ: Relative quantity, (BSI): bloodstream infection, (CT): catheter tip, (P): Pneumonia, (C): colonization and (PF): prosthesis fragment. ***Top right:*** The arrow indicates the arrow-tip-like zone of the δ-hemolysin activity on sheep blood agar. ***Bottom:*** Mean biofilm values (BU) for the populations formed by isolates showing hemolytic activity or absence of hemolysis.

**Figure 6:**
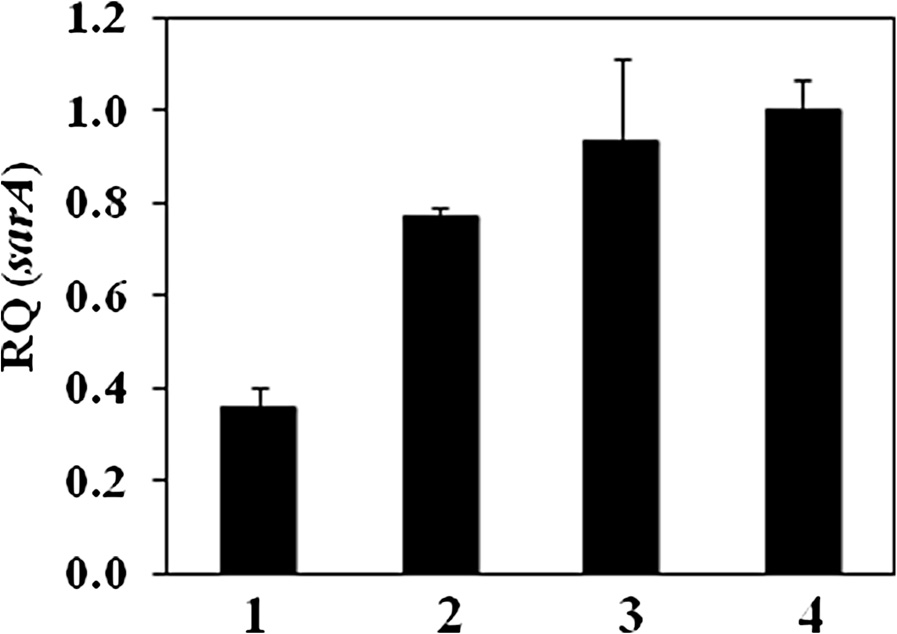
**Transcriptional levels of *sarA *determined by using ΔΔC**_**T **_**comparative method.** (**1**) USA400-related isolates 08–008 (*agr*-dysfunctional) and (**2**) 96/05 (*agr*-functional). (**3**) BMB9393 was used as a control and (**4**) RN6390B as calibrator. RQ: Relative quantity.

About 13% (8/60) of the USA400 related isolates exhibited no apparent hemolytic activity (Figure 5, top right). These 8 isolates had almost undetectable *agr* expression by RT-qPCR (Figure 5, top left). Of significance is the fact that 4 out of 8 *agr*-dysfunctional MRSA were recovered from BSI (50%). The RNAIII transcriptional levels for the 8 *agr*-functional isolates analyzed were significantly lower than that of strain RN6390B (Figure 5, top left). When we correlated the biofilm values (BU) with the levels of RNAIII transcription, we found that the population of clinical isolates with no hemolytic activity showed significant increase (*p*=0.01) in biofilm formation/accumulation (Figure 5, bottom). No significant difference could be detected in the values of oxacillin MIC when *agr*-functional (MIC_90_ = 128µg/mL) were compared with *agr*-dysfunctional isolates (MIC_90_ = 128µg/mL). Indeed, when we quantified *mecA* transcripts for 5 ST1 isolates, 08–008 (RQ=0.06±0.004), 89/05 (RQ=1.194±0.1), 08–068 (RQ=2.841±0.816), 07–135 (RQ=1.867±0.69), 07–058 (RQ=1±0.62), displaying different levels of *agr* expression (Figure 5, top left), we could not find a negative linear correlation between *mecA* and *agr* expressions (correlation coefficient, r = 0.823). Thus, an overexpression of *mecA* can not to be implicated in the inhibition of RNAIII transcription. Because *agr* is positively regulated by SarA, the expression of *sarA* gene was also analyzed by RT-qPCR. Our data showed a significant (*p*=0.0052) attenuation of *sarA* for the *agr*-dysfunctional isolate 08–008 when compared with the *agr*-functional 96/05 (Figure 6).

### Animal model

**Figure 7:**
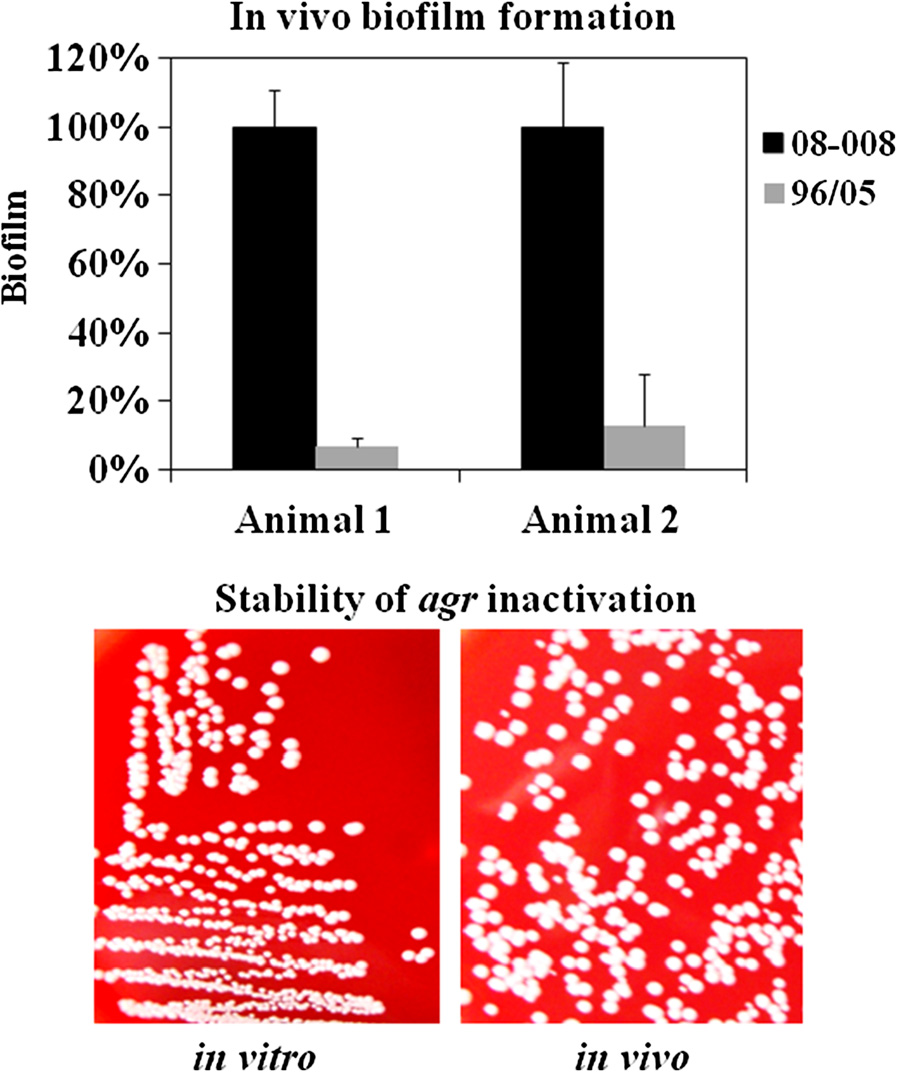
***In vivo *biofilm accumulation and stability of *agr *inhibition. *Top:*** For the foreign body animal model, data were transformed in percentage considering the CFU/mL of the isolate 08–008 as the reference value (100%). ***Bottom:*** The stability of *agr* inhibition was tested by examining the hemolytic activity of individual colonies of the isolates 08–008 before (***left***) and after (***right***) passage in the animal.

The naturally *agr*-dysfunctional MRSA was able to colonize and grow on the surface of implanted catheter fragment, as well as to accumulate an increased amount of biofilm (2-log CFU/mL) when compared with the *agr*-functional isolate (Figure 7, top). The stability of the *agr* expression in the *agr*-dysfunctional MRSA was examined by observing the hemolytic activity of individual colonies. No hemolytic halo was detected before and after passages in mice (Figure 7, bottom).

### Expression of *agr*-regulated genes

**Figure 8:**
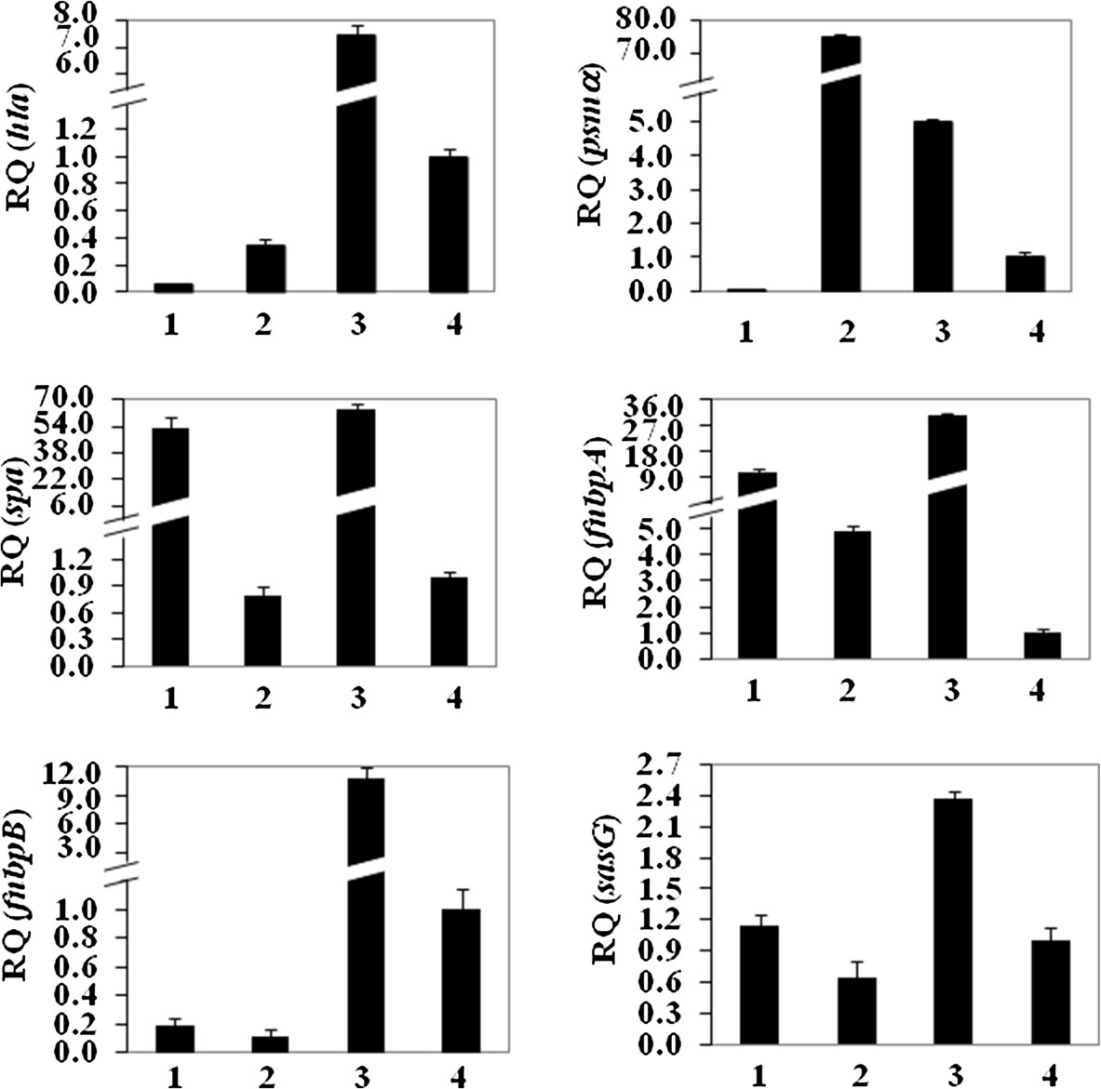
**Transcriptional levels of virulence-associated genes determined by RT-qPCR, using ΔΔC**_**T **_**comparative method.** (**1**) USA400-related isolates 08–008 (*agr*-dysfunctional) and (**2**) 96/05 (*agr*-functional). (**3**) BMB9393 was used as a control and (**4**) RN6390B as calibrator. RQ: Relative quantity.

Total RNA obtained from isolates with significant differences (*p*<0.001) in the RNAIII transcription level (08–008; RQ=0.0001±0.16 and 96/05; RQ=0.53±0.13) was used to analyze the expression of genes that are well known to be regulated by *agr*. As expected, the *agr*-up-regulated *hla* was less expressed (*p*<0.01) in the isolate 08–008 (Figure 8) when compared with the isolate 96/05 (RQ=0.05±0.01 and RQ=0.33±0.05, respectively). Similar pattern of expression was found for another *agr*-up-regulated gene, *psmα* (RQ_96/05_=75.90±0.10 and RQ_08-008_=0.005±0.12; *p*<0.001), except that in this case we also observed a very high expression of *psmα* for 96/05 (Figure 8). To verify if this amplified expression was a characteristic of this MRSA clone, other *agr*-functional isolates were randomly selected for testing. High level of *psmα* transcripts was also detected for the isolates 07–035, 07–059 and 08–068 (RQ_07 035_=35.71±0.06; RQ_07-059_=48.90±0.07; RQ_08-068_=31.30±0.07). For all virulence genes tested, the expression of the *agr*-functional isolate BMB9393 was higher than that of USA400-related isolates, except for *psmα* gene (Figure 8). Accordingly, the RNAIII-down-regulated *spa* gene showed a very significant lower expression (*p*<0.001) in the *agr*-functional 96/05 (RQ=0.8±0.20) compared with the *agr*-dysfunctional isolate 08–008 (RQ= 52.8±0.17; Figure 8).

### Expression of biofilm-associated genes *fnbAB*, *sasG* and *spa*

The *agr*-dysfunctional isolate 08–008, which showed increased biofilm accumulation *in vitro* and *in vivo*, had a significant increase (*p*=0.02) in *fnbA* transcripts (RQ_*fnbA*_=10.08±0.18) when compared with the isolate 96/05 RQ_*fnbA*_=4.91±0.19; Figure 8). However, no significant difference was detected when *fnbB* expression were analyzed (RQ_96/05_ =0.11±0.04; RQ_08-008_ =0.18±0.05; Figure 8). Similarly to *fnbA*, the expression of *sasG* (Figure 8; *p*=0.03) and *spa* (Figure 8; *p*<0.001) was also increased in 08–008 (RQ_*sasG*_=1.13±0.11; RQ_*spa*_=52.8±0.17) compared with 96/05 isolate (RQ_*sasG*_=0.65±0.14; RQ_*spa*_=0.8±0.20).

### Adherence and invasion

**Figure 9:**
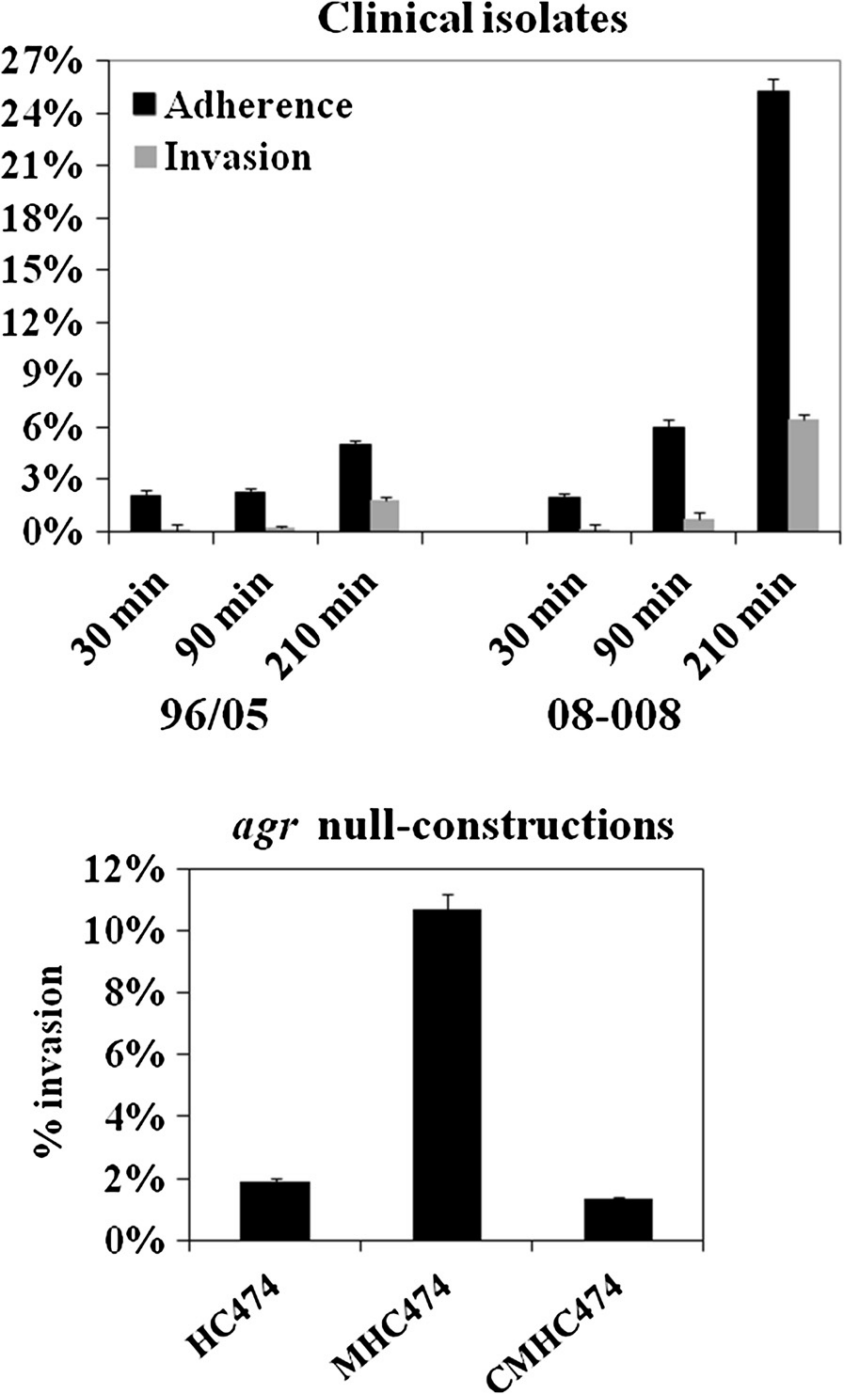
**Adherence and invasion assays using human bronchial epithelial cell line (16HBe14o**^**-**^**). *Top:*** 96/05 (*agr-*functional) and 08–008 (*agr*-dysfunctional). ***Bottom:*** Invasion assay was also determined after 3h30 min for the wild-type strain HC474, isogenic *agr* knockout MHC474 (Δ*agr*::*tetM*) and the *rnaIII*-trans-complemented construction CMHC474 (Δ*agr*::*tetM*, p*bla*-*rnaIII*).

The naturally *agr*-dysfunctional isolate 08–008 showed significant increase (*p*<0.05) in the adherence to human airway cells, reaching 25.27%±0.4% at 3h30min of incubation. In contrast, at the same conditions, the adherence of the *agr*-functional (isolate 96/05) to airway cells occurred in much less extent (4.94%±0.2%). Similarly, invasion was also higher for the *agr*-dysfunctional isolate (6.37%±0.3%) when compared with the *agr*-functional (1.76%±0.2%) at 3h30min incubation (Figure 9, top). Likewise, an increased invasive ability in the stationary phase was observed for the *agr*-knockout MHC474 (10.6%±0.3%) when compared with the wild type (HC474; 2.8%±0.1%) and complemented construction CMHC474 (2.3%±0.1%; *p*=0.0033; Figure 9, bottom).

## Discussion

The great majority of the USA400-related isolates (50/60; 83.3%) were able to accumulate strong/moderate biofilms on polystyrene surfaces. The isolates remaining produced weak biofilms. The ability to accumulate biofilm increased when the surfaces were covered with human fibronectin, as also reported by others [19,29]. In opposition to our results, it was reported that MW2 MRSA had a weak biofilm phenotype [30,31]. Similarly, a slight biofilm accumulation (OD=0.25-0.3) was observed for another USA400 strain called BAA-1683 [32]. In addition, recent data from our laboratory (Ramundo MS & Figueiredo AMS, 2012; unpublished observations) showed that another SCC*mec*IV isolates (ST30 CA-MRSA) accumulated much lower amount of biofilm compared with ST1-SCC*mec*IV isolates.

Previous data from our group [12] have also demonstrated that the ST1 isolates from Rio de Janeiro do not carry *lukSF* genes and have acquired a number of antimicrobial resistance traits. Thus, it is possible that the enhanced ability to accumulate biofilm, associated with the biological cost of acquired resistance and the absence of PVL, might have been the results (at least in part) of the microevolutionary events that accounted for changes in a previously community pathogen, promoting enhanced bacterial fitness to spread in hospitals and cause health-care associated diseases. The *ica*-independent nature of the biofilm formed by USA 400-related isolates was revealed by the disruption of bacterial film by proteinase K. Similar results were also observed by others using different MRSA isolates [33,34]. Some researchers have suggested that the bacterial autolysis increases eDNA concentration and, consequently, enhances the level of biofilm accumulation [20]. In fact, in our study, we observed a moderate correlation between biofilm accumulation and autolysis. In addition, we detected threefold increase in eDNA for the ST1 MRSA displaying enhanced ability to accumulate biofilm. Indeed, the addition of DNase I (56U/Well) caused a significant reduction (about 30%) in biofilm accumulation, suggesting eDNA cooperatively contributes to the biofilm architecture of ST1 isolates.

The statistical analysis showed that the group of clinical isolates with no hemolytic activity (*agr*-dysfunctional) had significant increase in the level of biofilm accumulation when compared with *agr*-functional isolates. These data are in agreement with previous studies for *agr*-laboratory knockouts [27,35,36], which have indicated that some *agr* mutants can display increased levels of biofilm accumulation. In spite of that, using another *S. aureus* strain it was reported that inhibition of *agr* reduced biofilm accumulation significantly [24,25]. In fact, *agr*RNAIII is a negative regulator of different surface proteins [22,23], and consistent with this regulation, amplified expression of genes encoding for biofilm-associated proteins FnBPA, SasG and Spa was found for the *agr*-dysfunctional variant. Both FnBPA and B have been implicated as major proteins for biofilm formation/accumulation in *S. aureus*[19,33]. However, despite the detection of an enhanced expression of *fnbA*, we could not find a significant increase in the transcription of *fnbB-mRNA* for the *agr*-dysfunctional ST1-MRSA. Equally, a study from Wolz and collaborators suggested that *fnbB* was not significantly affected by *agr*[36].

Confirming the *agr* inhibition detected, the expression of two genes up-regulated by RNAIII, *hla* and *psmα*, was lower compared with the *agr*-functional MRSA. Both cytolysins (HLA and PSM*α*) seem to have remarkable roles in the pathogenesis of *S. aureus*. HLA has been associated with lethal pneumonia in USA400 and USA300 strains [37,38]. It was also previously found that *psmα*-deleted mutant of CA-MRSA exhibited attenuated virulence in animal models [39]. In this study, we detected a superior expression of *pmsα* by the *agr*-functional isolates of USA400-related clone detected in Rio de Janeiro. In fact, it was shown by others that the transcription of *psmα-*mRNA was increased in most prevalent CA-MRSA lineages, including MW2, compared with other *S. aureus* isolates [39]. However, the molecular mechanisms involved with the enhanced expression of PSMα were not clarified [39]. Despite the importance of these virulence factors for *S. aureus* pathogenicity, it is remarkable that among the *agr*-dysfunctional variants, 4 were recovered from cases of BSI, 2 from colonization, 1 from pneumonia and 1 from infected prosthesis, showing that these variants were able to colonize and cause both severe acute (pneumonia and BSI) and chronic (foreign-body infection) staphylococcal diseases in humans. These data demonstrated that regardless the reduced virulence of *agr*-laboratory knockouts in some animal models [40], the virulence of naturally dysfunctional *agr* variants was confirmed for hospitalized patients. In contrast to the assumption that *agr*-dysfunctional isolates may not be able to initiate infections [41], the isolate 08–008 was able to colonize polyurethane endovenous catheter in a foreign-body mouse model, forming a denser biofilm accumulation when compared with the *agr*-functional isolate. It is important to state that because the ST1 isolates studied were not isogenic, it is possible that factors other than the inhibition of *agr* might also have accounted for the increased biofilm accumulation observed. Nevertheless, supporting our data, similar increase of the biofilm formed on catheters implanted in mice was previously reported for an *agr* laboratory knockout [28]. In opposition to the results obtained by Traber et al. [41], all individual colonies formed by the *agr*-dysfunctional MRSA remained non-hemolytic before and after passages in mice, strongly suggesting the genetic stability of the phenotype. This stability was confirmed for all *agr*-dysfunctional isolates from our collection. Corroborating our findings, while we were finishing this manuscript, we noticed the work by Park et al. [42] that found *agr* dysfunction in *S. aureus* significantly associated with persistent bacteremia with eradicated foci, even though the predominant MRSA isolates showed SCC*mec*II, *agr*II (possible belonging to USA100-New York/Japan clone) while the isolates studied here displayed SCC*mec*IV, *agr*III and clustered in USA400-MW2/WA-1 clone. In fact, the bacterial ability to adhere to and invade epithelial cells, and consequently evade host defense mechanisms, has already been associated with persistence in host cells and development of disseminated infections [43,44]. In the present study, the differential expression of *agr*RNAIII in MRSA clinical isolates had a significant impact on adherence and invasion at 3h30min incubation. The same impact was observed for the *agr* isogenic knockout, as previously showed by others using different cell lines and mostly laboratory mutants [26,45].

Recently, Pozzi et al. demonstrated that high level of PBP2a expression by the homogeneous methicillin-resistant derivative of the strain 8325–4 induced a proteinaceous biofilm and significant repression of the *agr* locus [46]. In addition, excision of the SCC*mec* element from the MRSA strain BH1CC, with consequent loss of oxacillin resistance, had the opposite effect on biofilm and lead to an increase of the *agr*RNAIII transcription. In addition, Rudkin et al. showed that methicillin resistance reduced the virulence of HA-MRSA by interfering with *agr*[47]. The great majority of ST1 isolates studied had MIC of 128 µg/mL (*agr*-functional or -dysfunctional), which is compatible with heterogeneous resistance to this drug. Indeed, *mecA* overexpression was not detected in the *agr*-dysfunctional isolates tested. SarA, a global transcriptional regulator of *S. aureus*, was previously found to be a positive regulator of *agr* and of biofilm formation/accumulation [21,48]. Thus, aiming to understand the mechanism involved in *agr* impairment in these clinical isolates, the level of *sarA* transcripts was also examined. It was observed that *sarA* expression was significantly diminished in the *agr*-dysfunctional compared with the *agr*-functional MRSA, suggesting the defect was upstream *agr*. Beeken et al. indicated that *sarA* repression inhibited biofilm accumulation due to SarA inhibition of both proteases and nucleases activity either in the presence or absence of *agr* mutations [49]. In contrast, the results obtained here demonstrated that *agr*-dysfunctional isolates showed increased biofilm accumulation, despite the fact that *sarA*-mRNA transcripts were reduced. In fact, other studies have showed that *sarA* or *agr*-*sarA* laboratory mutants had lower ability to bind to fibronectin due to *sarA* down-regulation of *fnbA* transcription [36]. Possible explanations for this apparent divergence could be the fact that the *agr*-dysfunctional ST1 studied showed only partial *sarA* inhibition, or may display strain-dependent variation in the genetic background affecting other genes apart to those studied.

## Conclusion

Isolates of this novel hospital-associated USA400 clone were able to accumulate moderate/strong amount of biofilms, *in vitro* and *in vivo*, and could efficiently adhere to and invade human airway cells. Moreover, *agr* inhibition was an ordinary phenomenon among those isolates, which seems to have impacted the expression of some important virulence genes studied. Although it is difficult to interpret *in vitro* studies in the light of what occurs in an infected human host, it follows logical that the enhanced adhesive properties combined with the acquisition of multiple drug resistance traits by ST1 isolates could have provided fitness advantages for spreading in hospital environments. Indeed, *agr*-dysfunctional isolates were recovered from cases of hospital pneumonia, bacteremia and infected prosthesis. Finally, our results strongly suggest that strategies for controlling MRSA biofilm based on *agr* inhibition approaches are unlikely to be effective, at least for ST1 MRSA isolates.

## Methods

### Isolates

Sixty USA400-related isolates were obtained from patients located in different hospital wards in Rio de Janeiro as part of standard clinical care. Thirty isolates were recovered from BSI (50%) and 8 from catheter tips (CT; 13.3%). The remaining were from colonization (C; 13.3%), pneumonia (P; 6.7%), skin/soft tissue infections (SSTI; 5%), urinary tract infections (UTI; 3.3%) and prosthesis fragment (PF; 1.7%). The infection sites had not been reported for 4 isolates. The *agr*-knockout MNY474 (Δ*agr*::*tetM*) and the *rnaIII*-trans-complemented mutant CMNY474 (Δ*agr*::*tetM*, p*bla*-*rnaIII*) were previously constructed from the clinical *S. aureus* isolate NY474 [27]. BMB9393 (ST239-SCC*mec*III) was used as positive control for biofilm and gene expression experiments [27]. The *S. aureus* RN4220 and RN6390B, a gift from Richard Novick (New York University), were used for hemolytic activity and gene expression analyses; respectively. This study was approved (#1055/09) by the Human Research Ethics Committee from Federal University of Rio de Janeiro, RJ, Brazil.

### Minimal inhibitory concentration (MIC)

Oxacillin MIC was determined using Müller Hinton plates and performed in accordance with the Clinical Laboratories Standards Institutes (CLSI) guidelines [50].

### *In vitro* biofilm assay

For all 60 isolates, biofilm was tested using 96-well inert polystyrene microtiter plates (Nunclon; Nunc A/S, Roskilde, Denmark) as previously described [28]. The biofilm unit (BU) was defined as indicated by Amaral et al. [14] and the isolates were classified as non-producers (BU≤0.230), weak (BU>0.230 and ≤0.460), moderate (BU>0.460 and ≤0.920) or strong producers (BU>0.920), as suggested [14]. For 19 isolates, biofilm assays were also carried out on surfaces covered with human fibronectin (Merck; Darmstadt, Germany) as previously described [28].

In some experiments, before treatment with crystal violet, the biofilm was treated with sodium metaperiodate (10mM/well; Sigma; St. Louis, MO, USA) or proteinase K (6U/well, Invitrogen; Carlsbad, California, EUA) [27]. Confocal laser scanning microscopy (CLSM) was employed to record and contrast structural images of the biofilm as described [28]. eDNA was quantified in biofilm supernatants using Qubit® 2.0 Fluorometer (Invitrogen; Eugene, Oregon, USA), after ethanol precipitation. For some experiments, biofilms were formed in the presence of DNase I (28U/well or 56U/well Invitrogen; Carlsbad, California, EUA).

### Animal model

A pair of isolates showing differential *agr* expression (08–008, *agr*-dysfunctional, obtained from BSI and 96/05, *agr*-functional, from CT) was used. The mouse subcutaneous catheter implant model was described in detail by Ferreira et al. [28]. Briefly, two intravenous polyurethane catheter segments (C-UDLM-953J model; Cook Medical, Bloominaton, USA) were implanted in the back of each anesthetized young-adult BALB/c male mice. Infection was induced 24 h after the implantation procedure by injecting a mid-exponential growth phase culture (10^6^ CFU/10 µL) into the lumen of the implanted catheter segment. The animal was euthanized after three days post-infection, and the catheter segments were surgically removed to assess the biofilm by counting catheter-adherent bacteria by CFU determination. Three independent experiments were performed. The animal study was approved (#IMPPG013) by The Ethics Committee for Animal Care and Use from Federal University of Rio de Janeiro, RJ, Brazil.

### DNase activity

Difco™ DNase Test Agar (BD; Becton, Dickinson and Company, Sparks, USA) was used to screen 17 USA400-related MRSA, as recommended by the manufacturer.

### Autolysis assay

Autolysin activity was measured in 8 selected isolates as previously described [51], except that cells were grown in TSB 1% Glc.

### Hemolytic activity

The δ-hemolysin (Hld), encoded by the *hld* gene, is codified within the *rnaIII* region and, consequently, the detection of δ-hemolysin is an indicative of *agr* expression. Sixty USA400-related isolates were screened for hemolytic activity on sheep red blood (5%) agar plates (Plast Labor, RJ, Brazil) as previously described [52].

### Gene expression

For RNA preparations, bacterial cells grown in TSB (18h/37°C; 250 rpm) were obtained in the exponential phase (OD_600nm_ = 0.3) and in the stationary phase. Total RNA was prepared using the RNeasy Mini kit (Qiagen; Maryland, USA) and quantified by the Qubit 2.0 Fluorometer. The RNA quality was analyzed by running RNA-gel electrophoresis. The real-time quantitative PCR (RT-qPCR) was carried out using Power SYBR® Green RNA-to-C_T_^TM^ 1-Step Kit (Applied Biosystems; Foster city, CA, USA) as recommended, using ΔΔCt comparative method. The primers and run conditions used for *rnaIII, hla, psmα*[53]*, sarA, mecA*[54]*, spa, sasG, fnbA* and *fnbB* genes and for the endogenous control *rrna* 16S are listed in Table 1. All primers designed for this study were validated as recommended (Guide to Performing Relative Quantitation of Gene Expression Using Real-Time Quantitative PCR; Applied Biosystems). The run was performed in the Step One™ Real Time PCR System (Applied Biosystems). Data were analyzed using the Step One Software 2.2 (Applied Biosystems).

**Table 1:** Primers used in Real Time qPCR

**Target gene**	**Primer sequence**^**a**^	**Amplicon length (bp)**	**Reference**
*rnaIII*	F: AATTTGTTCACTGTGTCGATAAT	135	This study
	R:TGGAAAATAGTTGATGAGTTGTT		
*sarA*	F: TTCTTTCTCTTTGTTTTCGCTG	115	This study
	R: GTTATCAATGGTCACTTATGCT		
*spa*	F: TGGTTTGCTGGTTGCTTCTTA	116	This study
	R: GCAAAAGCAAACGGCACTAC		
*hla*	F: TTTGTCATTTCTTCTTTTTCCCA	169	This study
	R: AAGCATCCAAACAACAAACAAAT		
*psmα*	F:TATCAAAAGCTTAATCGAACAATTC	176	53
	R: CCCCTTCAAATAAGATGTTCATATC		
*sasG*	F:GGTTTTCAGGTCCTTTTGGAT	192	This study
	R:CTGGTGAAGAGCGAGTGAAA		
*fnbpA*	F: ACTTGATTTTGTGTAGCCTTTTT	185	This study
	R:GAAGAAGCACCAAAAGCAGTA		
*fnbpB*	F:CGTTATTTGTAGTTGTTTGTGTT	118	This study
	R:TGGAATGGGACAAGAAAAAGAA		
*rrna* 16S	F: AGAGATAGAGCCTTCCCCTT	84	This study
	R:TTAACCCAACATCTCACGACA		
*mecA*	F:TCCAGATTACAACTTCACCAGG	162	54
	R:CCACTTCATATCTTGTAACG		

^a^F and R: forward and reverse primers, respectively, in 5´→ 3´orientation. The cycling conditions for all primers were as follows: One cycle of 48°C/30min and 95°C/10 min, followed by 35 cycles of 95°C/30s, 55°C/45s and 72°C/45 s. Each run included a nontemplate and a gene-negative RNA controls.

### Adherence and invasion kinetics

Bacterial adherence and invasion were investigated using human bronchial epithelial cells (16HBE14o^-^ cell line) as described [14], except that monolayers were prepared using Dulbecco´s Modified Eagle Medium (DMEM, Low Glucose 1X; Gibco, Invitrogen, Grand Island, USA) and 10% Fetal Bovine Serum (Gibco, Invitrogen). For determining the colony forming units (CFU) of the total adhered and invasive bacteria (CFU_AI_), infected monolayers were washed twice in DMEM (to remove non-adherent bacteria), incubated (5 min/37°C) with 0.25% (wt/vol) trypsin (11,000 U/mg; Sigma; St. Louis, MO USA), lysed (5 min/37°C) with 0.025% (vol/vol) Triton X-100 (Sigma) and plated in TSA. For determining the CFU of invasive bacteria (CFU_I_), infected monolayers were washed twice in DMEM and incubated (20 min/37°C) with 100 µg/mL lysostaphin (500 U/mg; Sigma) to lyse adherent bacteria. Monolayers were washed twice and incubated (5 min/37°C) with 0.25% (wt/vol) trypsin. The epithelial cells were lysed (5 min/37°C) with 0.025% (vol/vol) triton X-100 and plated. For each aliquot, the total CFU in the supernatant was also determined (CFU_S_). The CFU of adherent bacteria (CFU_A_) was obtained by the formula: CFU_A_ = CFU_AI_ - CFU_I_. The percentages of invasive or adherent bacteria were calculated considering as 100% the total CFU obtained by the sum of CFU_AI_ + CFU_S_ for each aliquot. In addition to the USA400-related isolates, the wild-type HC474, and the isogenic Δ*agr*::*tetM* and *rnaIII*-trans-complemented constructions were also used for investigating bacterial invasion.

### Statistical calculations

Student’s *t*-test (unpaired data) was used to compare the means of the biofilm values and of the data from gene expression experiments. In addition, correlation coefficient (r) was used to test the relationship between the autolysis and the ability of ST1 isolates to accumulate strong or weaker biofilms. This last test was also used to determine the occurrence of linear correlation between *mecA* and *agr* expressions [55]. Data were expressed in terms of mean values obtained from at least three independent experiments and three repetitions of each set.

## Abbreviations

Agr: Accessory gene regulator; BSI: Bloodstream infection; BU: Biofilm unit; C: Colonization; CA-MRSA: Community-acquired Methicillin-resistant *Staphylococcus aureus*; CFU: Colony-forming unit; CLSM: Confocal laser scanning microscopy; CT: Catheter tip; DNase: Desoxyribonuclease; DMEM: Dulbecco´s Modified Eagle Medium; eDNA: Extracellular DNA; Fn: Fibronectin; fnbAB: Genes encoding for fibronectin-binding protein A and B; FnBPA/FnBPB: Fibronectin-binding protein A and B; ica: Operon encoding for enzymes involved in the synthesis of the polysaccharide of intercellular adhesion (PIA); HA-MRSA: Hospital-associated methicillin-resistant *Staphylococcus aureus*; Hla: α-Hemolysin; Hld: δ-Hemolysin; lukSF: Genes involved in the synthesis of the subunits S and F of the Panton Valentine leukocidin; MIC: Minimal inhibitory concentration; mecA: Gene encoding for penicillin-binding protein 2A; MRSA: Methicillin-resistant *Staphylococcus aureus*; OD: Optical density; P: Pneumonia; PBP2A: Penicillin-binding protein 2A; PCR: Polymerase chain reaction; PF: Prosthesis fragment; Psm: Phenol-soluble modulin; PVL: Panton-Valentine leukocidin; r: Correlation coefficient; Rot: Repressor of toxins; RQ: Relative quantity; RT-qPCR: Real time quantitative polymerase chain reaction; rrna 16S: Gene encoding for ribosomal RNA 16S; SarA: Transcriptional regulator SarA; SasG: *S. aureus* surface protein G; SCCmec: Staphylococcal cassette chromosome *mec*; ST: Sequence-type; Spa: Protein A; SSTI: Skin and soft tissue infections; TSB: Tryptic soy broth; TSB 1% Glu: Tryptic soy broth supplemented with 1% glucose; UTI: Urinary tract infections.

## Competing interests

The authors declare that they have no competing interests.

## Authors’ contributions

FAF wrote the draft paper and carried out the experiments of biofilm formation/accumulation on inert polystyrene surfaces, DNase activity, autolysis assay, hemolytic activity, gene expression experiments, DNA Sequencing and statistical calculations. RRS, MAA and SELF carried out experiments of the animal model including animal surgery and observation, and biofilm determinations. RRS also carried out oxacillin MIC determinations. BSM carried out the experiments of biofilm formation/accumulation on inert polystyrene surfaces and also on implanted catheters. AMAF and JNS carried out studies of adherence and invasion kinetics. AMSF carried out the experiments on *mecA* gene expression and was responsible for the study design, methodology used, wrote and review the draft paper and gave final approval of the manuscript. All authors read and approved the final manuscript. All authors contributed significantly for the conduction of the studies and discussion of the results.
